# Assessment of the current state of biodiversity data for butterflies and skippers in the state of Mato Grosso, Brazil (Lepidoptera, Papilionoidea and Hesperioidea)

**DOI:** 10.3897/zookeys.595.7856

**Published:** 2016-06-03

**Authors:** Luziany Queiroz-Santos, Fernando Maia Silva Dias, Rafael Dell’Erba, Mirna Martins Casagrande, Olaf Hermann Hendrik Mielke

**Affiliations:** 1 Laboratório de Estudos de Lepidoptera Neotropical, Departamento de Zoologia, Universidade Federal do Paraná, Caixa Postal 19.020, 81.531-980, Curitiba, Brazil; 2 Museu de Zoologia, Universidade de São Paulo, Av. Nazaré, 481, 04263-000, São Paulo, Brazil

**Keywords:** Biodiversity, Database, Amazon, Cerrado, Pantanal, Occurrence

## Abstract

Lepidoptera is one of the four megadiverse insect orders, comprising butterflies and moths. In Brazil, the bulk of knowledge about the butterfly fauna is restricted to some areas in the southeast of the country, with large gaps of knowledge in other areas. The state of Mato Grosso is one of the largest states in Brazil, and holds three of the main Brazilian biomes: Amazon rain forest, Cerrado and Pantanal. However, knowledge about Mato Grosso butterflies is fragmented and restricted to a few localities, and information is scattered in various sources. The aim of this study is to assemble the biodiversity information of the butterfly fauna of the state of Mato Grosso based on historical and recent literature data and collections carried out in the southwest of the state from 2007–2009. Records without precise locality data or taxonomic information were not included. Species identification was based on literature and comparison with specimens in collections; higher and species-level taxonomy were updated based on the Neotropical Checklist of Hesperioidea and Papilionoidea and recent phylogenetic and revisionary taxonomic works. In total, 901 species were recorded in 2,820 occurrence records. This represents 148 species of Hesperiidae, 29 Papilionidae, 28 Pieridae, 77 Lycaenidae, 238 Riodinidae, and 381 Nymphalidae. Of these, 207 species records are from the type specimens of species described in the state. Based on the results and literature records for other Brazilian states and biomes, probably the figures for Mato Grosso are underestimated, particularly in the families Hesperiidae, Lycaenidae and Riodinidae, in that order. Future collecting efforts should be directed towards certain areas of the state, especially in less sampled areas and biomes, as the north of the state and Pantanal.

## Introduction

The butterflies are a highly suitable taxonomic group for assessing environmental disturbance and its impact on species conservation. They are effective biodiversity indicators; similarly, their charismatic appeal and biological peculiarities make them an effective “umbrella group” for biodiversity and habitat conservation (Brown Jr 1992, Brown Jr and Freitas 1999, Santos et al. 2008).

Gathering species distribution data is essential for any practical decision about species conservation (Lewinsohn and Prado 2002). However, even though interest in the conservation of biodiversity has recently increased, species inventories and lists are still lacking (Mielke et al. 2008), and the bulk of the knowledge about the butterfly fauna of Brazil is restricted to a few areas in the southeast of the country, with large gaps of knowledge in other areas (Santos et al. 2008). Along with many other organisms, butterflies are threatened by the destruction and fragmentation of their natural habitats (Emery et al. 2006) and therefore efforts to gather local and regional species lists should be intensified before natural habitats have been altered by anthropic landscapes (Lewinson and Prado 2005).

The state of Mato Grosso is potentially highly biodiverse because three of the main Brazilian biomes are present within its borders: Amazon tropical rainforest, Cerrado and Pantanal. However, knowledge about Mato Grosso butterflies is fragmentary and information is scattered throughout various sources (Santos et al. 2008). Given the absence of comprehensive regional lists for most of the Brazilian states, the aim of this study is to accrue and present the biodiversity data for the butterfly fauna in the state of Mato Grosso, based on literature data from the years 1895 to 2015, and collections carried out in the southwest region of the state from 2007 to 2009. Additionally, records assigned to the state of Mato Grosso in error are corrected.

## General description

**Additional information**: The authors would like to thank Marcelo Duarte (MZUSP) and Geraldo Lamas (UNMSM) for providing access to essential literature, Marcelo Medaglia for help with maps and georeferencing, and Fábio Santos for suggestions about the manuscript and Keith M. Bayless for reviewing the English version of the manuscript. We also would like to thank the following researchers for help in the identification of specimens: Alfred Moser (Lycaenidae), Eduardo Carneiro (Hesperiidae), Diego Dolibaina (Riodinidae), Thamara Zacca (Satyrinae), Eduardo Barbosa for species of Hermeuptychia, and Lucy Mila Salik (Biblidinae). The authors would like to thank the Conselho Nacional de Desenvolvimento Científico e Tecnológico (CNPq) and Coordenação de Aperfeiçoamento de Pessoal de Nível Superior (CAPES) for the fellowships granted to the authors (LQS: 130624/2014-1, CNPq; FMSD: Edital 15/2014, CAPES/EMBRAPA; RD: CAPES; MMC: 308247/2013-2, CNPq; OHHM: 304639/2014-1, CNPq).

## Project details

**Project title**: Assessment of the current state of biodiversity data for butterflies and skippers (Lepidoptera: Papilionoidea and Hesperioidea) in the state of Mato Grosso, Brazil

**Personnel**: Luziany Queiroz-Santos, Fernando Maia Silva Dias, Rafael Dell’Erba, Mirna Martins Casagrande, Olaf Hermann Hendrik Mielke

**Funding**: Conselho Nacional de Desenvolvimento Científico e Tecnológico (CNPq), Coordenação de Aperfeiçoamento de Pessoal de Nível Superior (CAPES)

**Study area descriptions/descriptor**: Mato Grosso is located in the central-western part of Brazil, with an area of 903,378,292 km², making it the third largest state in Brazil. The state has three different climate zones: in lower elevations, there is a tropical monsoon climate, with rainy summers and dry winters and an average temperature of over 24°C; and also a tropical rainforest climate, with no distinct seasons, heavy rainfall, and average temperature of 23°C; and in higher elevations, there is a subtropical climate, with an average temperature of 17°C. Most regions are at low to medium elevations, with areas from about 100 meters in the southwest and northern areas, reaching up to 1,118 meters above sea level; nevertheless, about two thirds of the state is below 600 meters in elevation. Mato Grosso is drained by streams that flow north to the Amazonas drainage basin (e.g. Juruena, Teles Pires, and Xingu rivers), east to the Tocantins-Araguaia river basin (e.g. Araguaia River), and south to the Paraná river basin (e.g. Cuiabá River).

**Design description**: The list of diurnal butterflies occurring in the state of Mato Grosso, Brazil, was compiled based on faunistic studies, species descriptions and other taxonomic literature, and specimens collected by the first author in field expeditions carried out between November 2007 and January 2009 in the municipality of Pontes e Lacerda, southwestern Mato Grosso.

The first records of butterflies from the state of Mato Grosso are the type localities of species described by Fruhstorfer (1895) and Godman and Salvin (1896); in the following years, several authors added records to the state. The Talbot and Collenette expedition was the first significant contribution to the knowledge of the butterfly fauna of Mato Grosso; two papers, both published in 1928, provide several records and descriptions of new taxa. The results of the Rondon expedition led by Miranda-Ribeiro (1931), records provided by Brown Jr. (1979, 1987), and data available in the illustrated guide by Garwood et al. (2009), stand out as important sources of occurrence records. A total of 2,820 individual occurrence records were included in the database; of the 901 species recorded, 207 are from type localities of descriptions of taxa and 102 from the three years of field work in the municipality of Pontes e Lacerda. Of these, 31 species were not recorded before in the literature to Mato Grosso. The highest number of records are from Nymphalidae (381 species in 1,669 records), followed by Riodinidae (238 species in 605 records) and Hesperiidae (148 species in 177 records). The families with the least number of both species and records are Lycaenidae (77 species in 105 records), Pieridae (28 species in 151 records) and Papilionidae (29 species in 94 records).

## Data published through

GBIF: http://ipt.sibbr.gov.br/sibbr/resource?r=ufpr_borboletasmt

## Taxonomic coverage

**General taxonomic coverage description**: The taxonomic coverage of this dataset spans the diurnal butterflies, which includes superfamilies Hesperioidea (with one family, Hesperiidae) and Papilionoidea (with five families, Papilionidae, Pieridae, Nymphalidae, Lycaenidae and Riodinidae). The highest number of records in the study area are from Nymphalidae (381 species in 1,669 records), followed by Riodinidae (238 species in 605 records) and Hesperiidae (148 species in 177 records). The families with the least number of both species and records are Lycaenidae (77 species in 105 records), Pieridae (28 species in 151 records) and Papilionidae (29 species in 94 records).

## Taxonomic ranks

Kingdom: Animalia

Phylum: Arthropoda

Class: Insecta

Order: Lepidoptera

Family: Papilionidae, Pieridae, Riodinidae, Nymphalidae, Lycaenidae, Hesperiidae

**Common names**: Animals, Arthropods, Insects, Butterflies and Moths, Swallowtails, Sulphurs and Whites, Metalmarks, Brush-footed Butterflies, Blues, Coppers and Hairstreaks, Skippers

## Spatial coverage

**General spatial coverage**: This dataset collates species occurrences from the Brazilian state of Mato Grosso. Most regions are at low to medium elevations, with areas from 100 meters in the southwest and northern limits, reaching up to 1,118 meters above sea level, however, about two thirds of the state is below 600 meters in elevation.

**Coordinates**: 18°7'12"S and 7°22'48"S Latitude; 61°36'0"W and 50°23'60"W Longitude

**Figure 1.**
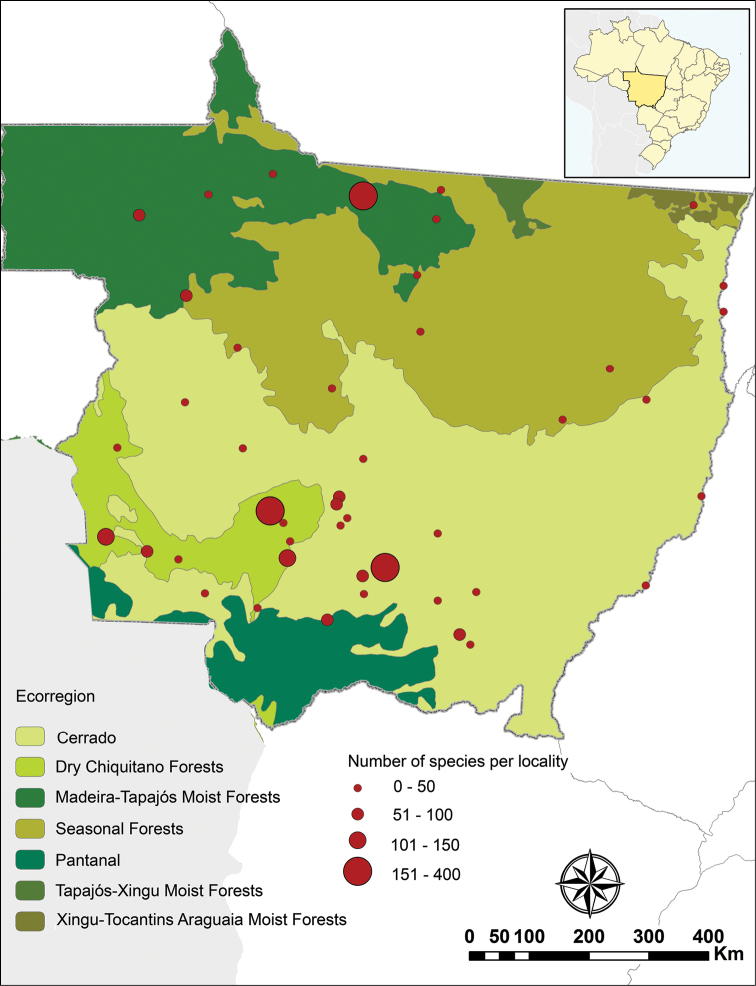
Occurrence localities of butterflies and number of species per locality in the state of Mato Grosso, Brazil.

**Temporal coverage**: 1895 - 2015

**Natural collections description**

**Collection name**: Coleção Zoobotânica “James A. Ratter”, Universidade do Estado de Mato Grosso, Nova Xavantina, Mato Grosso, Brazil

**Collection identifier**: CZNX

**Natural collections description**

**Collection name**: Coleção Entomológica Padre Jesus Santiago Moure, Universidade Federal do Paraná, Curitiba, Paraná, Brazil

**Collection identifier**: DZUP

**Specimen preservation method**: Mounted

## Methods

**Method step description**: Compilation of occurrence data from the literature, and data of the specimens collected between November 2007 and January 2009 in the municipality of Pontes e Lacerda, southwestern Mato Grosso.

**Study extent description**: Literature and specimens between November 2007 and January 2009 in the municipality of Pontes e Lacerda, southwestern Mato Grosso.

**Sampling description**: The list of diurnal butterflies occurring in the state of Mato Grosso, Brazil, was compiled based on faunistic studies, species descriptions and other taxonomic literature (Fruhstorfer 1895; Godman and Salvin 1896, Godman and Salvin 1898, Godman 1900, Godman 1903, Druce 1904, Godman 1905, Druce 1907, Stichel 1909, Fruhstorfer 1910a, Fruhstorfer 1910b, Niepelt 1910, Stichel 1910, Weymer 1911, Fruhstorfer 1912, Röber 1913, Fruhstorfer 1915, Stichel 1915, Fruhstorfer 1916a, Fruhstorfer 1916b, Oberthür 1916, Stichel 1916a, Stichel 1916b, Seitz 1917, Reverdin 1919, Stichel 1919, Lathy 1921, Riley 1921, Röber 1921, Skinner 1921, Martin 1923, Stichel 1923, Joicey and Talbot 1924, Riley 1924, Stichel 1924, Joicey and Talbot 1925, Neustetter 1925, Röber 1925, Lathy 1926a, Lathy 1926b, Stichel 1926, Williams 1926, Williams 1927, Collenete and Talbot 1928a, Collenette and Talbot 1928b, D’Almeida 1928, Talbot 1928, Hall 1929, Riley 1929, Stichel 1929, Miranda-Ribeiro 1931, Lathy 1932, Le Moult 1932, Seitz 1932, Talbot 1932, D’Almeida 1935, Bell 1938, Williams and Bell 1939, D’Almeida 1941, Hayward 1942a, Hayward 1942b, Evans 1944, Goodson 1945, Dillon 1948, Evans 1951, D’Almeida 1952, Bryk 1953, Evans 1953, Evans 1955, D’Almeida 1958, Le Moult and Réal 1962–1963, Weber 1963, Mielke 1967, Mielke 1968, Brown Jr et al. 1970, Brown Jr 1973, Mielke 1978, Casagrande and Mielke 1979, Bristow 1981, 1982, 1991, De Jong 1983, Jenkins 1983, Brown Jr 1987, Steinhauser 1991, Blandin 1993, Johnson 1993, Burns 1994, Tyler et al. 1994, D’Abrera 1995, Mielke 1995, Hall and Willmott 1996, Johnson and Kruse 1997, Austin and Mielke 1997, Hall 1998, Callaghan 1999, Hall and Furtado 1999, Harvey and Hall 2002, Blandin 2007b, Garwood and Lehman 2009, Casagrande 2009, Dolibaina et al. 2013, Dorval et al. 2013, Kaminski et al. 2015). Only occurrences explicitly recorded in a locality within the limits of the state of Mato Grosso were acknowledged, therefore, approximately 470 species of Hesperiidae listed by Brown Jr (1987) were not recorded, as his list also contains data from neighboring states. Similarly, occurrences without precise taxonomic information (e.g. unidentified species, species noted with “cf.”, “aff.”, “?”, and uncertain identifications, when explicitly stated, etc.) were not included. Due to factual errors and successive changes in the political boundaries of the states of Brazil, some records of type specimens supposedly from “Mato Grosso” (Lamas 2004, Mielke 2005a,b,c,d,e,f) actually belong to the Brazilian states of Rondônia, Mato Grosso do Sul or Pará. The type localities of these species were corrected, based on the information provided in the original descriptions and assigned to the correct state. Additionally, data from specimens collected by LQS in field expedition carried out between November 2007 and January 2009 in the municipality of Pontes e Lacerda, Mato Grosso were included. Specimens were actively collected with standard entomological nets, mounted, labeled, identified, and deposited at the Coleção Zoobotânica “James A. Ratter”, Universidade do Estado de Mato Grosso, Nova Xavantina (CZNX).

Higher and species-level taxonomy of all records were checked and updated, based on Blandin (1988), McAlpine (1971), Jenkins (1985), Jenkins (1990), Bristow (1981, 1982, 1991), Holzinger and Holzinger (1994), Tyler et al. 1994, Hall (1998), Hall (2000), Willmott (2003), Lamas (2004), Bálint (2005), Hall (2005), Blandin (2007a), Hall (2007), Austin (2008), Penz (2008), Faynel et al. (2012), Dolibaina et al. (2013), Ortiz-Acevedo and Willmott (2013), Seraphim et al. (2013), Zacca et al. (2013), Dias et al. (2014) and Díaz et al. (2014). Collected specimens were identified through direct comparison with specimens deposited at the Coleção Entomológica Pe. Jesus Santiago Moure, Departamento de Zoologia, Universidade Federal do Paraná (DZUP) and with the aid of specialists (see additional information).

## Datasets

**Dataset description**

**Object name**: Darwin Core Archive Assessment of the current state of biodiversity data for butterflies and skippers (Lepidoptera: Papilionoidea and Hesperioidea) in the state of Mato Grosso, Brazil

**Character encoding**: UTF-8

**Format name**: Darwin Core Archive format

**Format version**: 1.0

**Distribution**: http://ipt.sibbr.gov.br/sibbr/archive.do?r=ufpr_borboletasmt

**Publication date of data**: 2016-03-30

**Language**: English

**Licenses of use**: This work is licensed under a Creative Commons Attribution Non Commercial (CC-BY-NC) 4.0 License (http://creativecommons.org/licenses/by-nc/4.0/legalcode).

**Metadata language**: English

**Date of metadata creation**: 2016-01-21

**Hierarchy level**: Dataset
